# A New Application of Functional Zonal Image Reconstruction in Programming for Parkinson's Disease Treated Using Subthalamic Nucleus–Deep Brain Stimulation

**DOI:** 10.3389/fneur.2022.916658

**Published:** 2022-06-10

**Authors:** Jiaming Mei, Bowen Chang, Chi Xiong, Manli Jiang, Chaoshi Niu

**Affiliations:** Department of Neurosurgery, The First Affiliated Hospital of USTC, University of Science and Technology of China, Hefei, China

**Keywords:** Parkinson's disease, deep brain stimulation, programming, subthalamic nucleus, image reconstruction

## Abstract

**Objective:**

Programming plays an important role in the outcome of deep brain stimulation (DBS) for Parkinson's disease (PD). This study introduced a new application for functional zonal image reconstruction in programming.

**Methods:**

Follow-up outcomes were retrospectively compared, including first programming time, number of discomfort episodes during programming, and total number of programming sessions between patients who underwent image-reconstruction-guided programming and those who underwent conventional programming. Data from 142 PD patients who underwent subthalamic nucleus (STN)-DBS between January 2017 and June 2019 were retrospectively analyzed. There were 75 conventional programs and 67 image reconstruction-guided programs.

**Results:**

At 1-year follow-up, there was no significant difference in the rate of stimulus improvement or superposition improvement between the two groups. However, patients who underwent image reconstruction-guided programming were significantly better at the first programming time, number of discomfort episodes during programming, and total number of programming sessions than those who underwent conventional programming.

**Conclusion:**

Imaging-guided programming of directional DBS leads was possible and led to reduced programming time and reduced patient side effects compared with conventional programming.

## Introduction

Parkinson's disease (PD) is a neurodegenerative syndrome involving multiple motor and non-motor neural circuits in the basal ganglia. Subthalamic nucleus (STN) deep brain stimulation (DBS) is an effective treatment for patients with advanced PD and motor complications (1–3). Common DBS targets for PD include the *globus pallidus pars interna* (GPi), the STN and, less often, the ventral intermediate nucleus of the thalamus. A recent review concluded that GPi-and STN-DBS provide similar and consistent benefits with subtle target differences (4, 5). Target selection should be tailored to each patient's clinical presentation. Numerous factors contribute to positive outcomes of DBS, including careful patient selection, lead placement, and effective programming (6). Only DBS programming can be modified after patient implantation; therefore, DBS programming plays a crucial role in improving clinical outcomes (7).

Nevertheless, for three decades, programming has remained a manual and time-consuming process that requires highly trained and experienced clinicians to achieve maximal therapeutic benefit in each patient (8, 9). Other sessions are often organized during follow-up visits to manage stimulation-induced side effects (e.g., speech problems and stimulation-induced dyskinesias) or worsening of the underlying parkinsonism. While the utility of these reprogramming sessions is well-established, no guidelines are available, and most of these changes rely on the results of a few open-label studies (10–12). In fact, although DBS has been used for almost three decades, systematic programming protocols remain lacking, leading to inconsistent and inefficient stimulation adjustments, as well as numerous or unnecessary patient visits. Our center used image reconstruction technology to reconstruct the nuclei and electrodes, and used this to guide programming and obtained satisfactory results.

## Methods

### Patients

This study and the STN-DBS protocol were approved by the Ethics Institutional Committee of the First Hospital affiliated with USTC (China). All patients provided informed consent to participate in the study. Records from 142 patients with PD undergoing STN-DBS, performed by the same surgeon between January 2017 and January 2021, were analyzed. Between January 2017 and June 2019, 75 patients comprising the control group underwent conventional programming, and 67 were guided to a program based on functional zonal image reconstruction after improved programming methods from June 2019 to January 2021.

### Image Reconstruction

First, imaging data from the patients were obtained, including postoperative computed tomography (CT; thin layer, 0.62 pitchless scan 5 mm) and preoperative localization magnetic resonance imaging (MRI; 3.0 Tesla, 2 mm pitchless scan). Next, the lead DBS was installed through the MATLAB platform and, after successful installation, imaging data were imported. Second, postoperative CT data were aligned with preoperative MRI data. Third, preoperative MRI data were standardized into the cranial model to obtain transformation parameters. Fourth, target reconstruction was performed. Finally, the electrode contact position was stimulated.

### Programming Process

Programming was not initiated immediately after surgery but 4 weeks later, when the initial microlesion benefits faded. At the appointed time, patients visited the outpatient clinic. Programming sessions were performed in the “OFF” medication state after the overnight withdrawal of all dopaminergic medications for at least 12 h. The pulse width was standardized to 60 ms and the stimulation frequency was set to 130 Hz for both DBS programming sessions. The physician was able to query and record patient medical history. The patients' motor symptoms were evaluated using the Unified Parkinson's Disease Rating Scale (UPDRS III, except for rigidity and postural stability). The physician placed the programmer close to the patient's skin surface where the stimulator had been implanted. After the programmer was connected to the stimulator, the physician was able to view current parameters, adjust parameters (including voltage, pulse width, frequency, stimulated contact, and electrode configuration adjustment), set limits of the patient programmer, start up and shut down the stimulator, and to check impedance. According to functional zonal image reconstruction, the electrode contacts located in the STN sensorimotor region were defined as the optimal contact of the image, and the optimal contact of the image was preferentially selected for programming. Programming without using functional zonal image reconstruction as guidance is referred to as conventional programming.

### Outcome Evaluation

All patients were assessed for PD severity using the UPDRS III drug on (i.e., with drugs), UPDRS III (without drugs), and UPDRS IV, while Mini-Mental State Examination (MMSE) and Montreal Cognitive Assessment (MoCA) scores were used to assess the cognitive status of the patients. The first programming time, discomfort episodes during programming, and total number of programming sessions were recorded. Discomfort during programming included dizziness, headache, blurred vision, numbness in the limbs, speech difficulties, and palpitation. Surgical outcome was assessed according to the stimulus improvement rate (UPDRS III score improvement compared to pre-operation when stimulated alone without the drug) and superposition improvement rate (UPDRS III improvement compared to pre-operation when stimulated with the drug) at 1 year after surgery.

### Statistical Analyses

All statistical analyses were performed using Empower(R) (www.empowerstats.com, X&Y Solutions, Inc., Boston, MA, USA) and R (http://www.R-project.org). Initially, the Kolmogorov–Smirnov test was performed to examine data distribution of the variables. Subsequently, data conforming to a normal distribution were evaluated using a two-tailed Student's *t*-test or one-way analysis of variance (ANOVA). Non-parametric data between different groups were compared using the Mann–Whitney test. Differences with two-tailed *P* < 0.05 were considered to be statistically significant.

## Results

Demographic data of the patients and scale scores were comparable between the two groups (Table 1). The mean (± SD) age of the control group was 59.17 ± 8.77 years and 59.37 ± 8.42 years for the image reconstruction group. As shown in Table 1, there were no significant differences in age, sex, duration, UPDRS III, UPDRS IV, MMSE, and MoCA scores between the control and image reconstruction groups.

**Table 1:** Characteristics of the patients with image reconstruction group and conventional programming group.

	**Conventional programming group**	**Image reconstruction group**	***P*-value**
Number of patients	75	67	
Age (years)	59.17 ± 8.77	59.37 ± 8.42	0.987
Duration (years)	8.01 ± 3.38	8.54 ± 3.78	0.590
Gender			0.916
Male	52 (69.33%)	47 (70.15%)	
Female	23 (30.67%)	20 (29.85%)	
UPDRS III med off	7.04 ± 1.53	6.99 ± 1.69	0.608
UPDRS III med on	21.85 ± 12.52	24.51 ± 11.64	0.195
UPDRS IV	6.83 ± 1.80	6.94 ± 1.58	0.691
MMSE	26.01 ± 3.28	26.33 ± 3.09	0.558
MoCA	20.88 ± 5.39	20.73 ± 5.31	0.869

In terms of surgical outcome, the mean stimulation improvement rate was 0.46 ± 0.15 in the control group and 0.40 ± 0.18 in the imaging reconstruction group—a difference that was not statistically significant. Similarly, the superposition improvement rate was 0.63 ± 0.15 in the control group and 0.64 ± 0.16 in the image reconstruction group, which was also not a significant difference (Table 2).

**Table 2:** Comparison of stimulus improvement rate, superposition improvement rate between conventional programming group and image reconstruction group at 1 year after surgery.

	**Conventional programming group**	**Image reconstruction group**	***P*-value**
Improvement rate med off	0.46 ± 0.15	0.40 ± 0.18	0.384
Improvement rate med on	0.63 ± 0.15	0.64 ± 0.16	0.978

**Figure 1:**
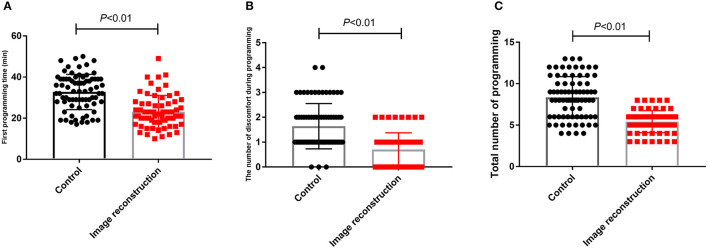
Comparison of first programming time **(A)**, number of discomfort episodes during programming **(B)** and total number of programming during 1 year after surgery **(C)** between control group and image reconstruction group.

Regarding programming, the first programming time was 32.77 ± 8.57 min in the control group and 23.15 ± 7.90 min in the image reconstruction group. The mean number of discomfort episodes during programming was 1.64 ± 0.91 in the control group and 0.70 ± 0.67 in the image reconstruction group. The total number of programming sessions was 8.34 ± 0.29 in the control group and 5.42 ± 0.16 in the image reconstruction group (Table 3). Therefore, the image reconstruction group exhibited obvious advantages in the first programming time, the number of discomfort episodes during programming, and the total number of programming sessions (Figure 1).

**Table 3:** Comparison of first programing time, number of discomfort during programming between conventional programming group and image reconstruction group.

	**Conventional programming group**	**Image reconstruction**	***P*-value**
First programing time(min)	32.77 ± 8.57	23.15 ± 7.90	<0.001
Number of discomfort during programming	1.64 ± 0.91	0.70 ± 0.67	<0.001
Total number of programming	8.34 ± 0.29	5.42 ± 0.16	<0.001

## Discussion

DBS is an established and effective treatment for PD. After electrode(s) implantation, connection wires are internalized and connected to an implantable pulse generator (IPG) in the upper chest. Patients then participate in a number of extensive programming sessions to define the best stimulation parameters for optimal symptom management. The aim of this study was to compare conventional clinical DBS programming with an individualized image reconstruction-based programming approach.

**Figure 2:**
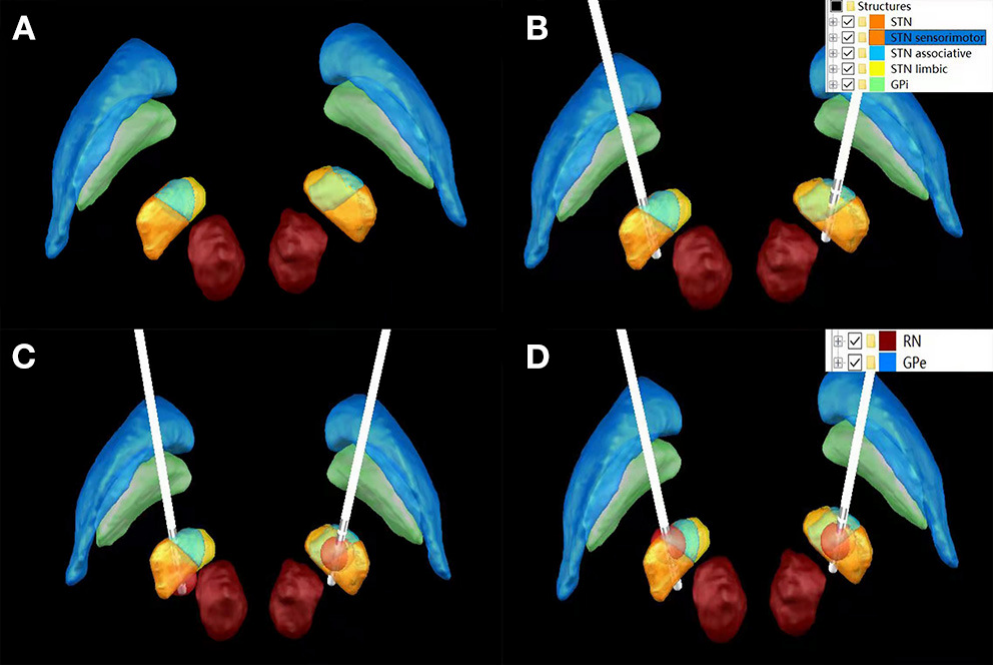
Bilateral STN morphology without Lead implantation **(A)**; Lead implantation on bilateral STN **(B)**; 1.5 V stimulation of K1 contact of left Lead involved sensorimotor, associative and limbic regions, while 1.5 V stimulation of K8 contact of left Lead only stimulated sensorimotor regions **(C)**; 1.5 V stimulation of K1 contact of lead on the left and K10 contact of lead on the right involved sensorimotor, Associative and limbic functional areas **(D)**.

The preferential target is the sensorimotor portion of the subthalamic nucleus (STN) which is often located within its dorsolateral part (13–15). Yet, the existence and location of a potential anatomical sweet spot within the STN remains a much debated question (16). Effective symptom control has been associated with active contacts being located around the dorsolateral border of the STN, indicating that not stimulation of the nucleus itself, but of adjacent white matter tracts might be accountable for symptom relief (17). Although STN discharges can be recorded by microelectrodes during operation, it is still impossible to distinguish the functional regions of STN from the microelectrode records. Therefore, postoperative image reconstruction of electrode and STN is helpful to guide postoperative stimulation contact selection and turn-on voltage for “visualization and predictability” guidance (Figure 2).

Despite accurate lead placement in the anatomical target, identification of optimal stimulation settings requires in-depth evaluation of all available contacts of the DBS lead and often even individualized settings for pulse frequency or width. Programming sessions may hence extend to several hours of time and be therefore exhausting for patients and clinicians, likewise. Furthermore, the evaluation of therapeutic and side effects of stimulation relies on high levels of training and experience of the performing clinician, making computer-based support highly desirable.

The need for further aid when it comes to DBS programming has been accentuated with modern DBS systems. While traditionally DBS leads consisted of four circular contacts, more sophisticated designs introduced lately to clinical routine allow further shaping of the electrical field achieving an increased therapeutic window (18, 19). This extension of the parameter space resulted, however, in an exponential increase of duration of clinical programming due to the almost uncountable potential parameter combinations. There have been considerable efforts to develop tools using imaging data to ascertain where stimulation might be most effective (20). Nevertheless, these advances have been restricted to a small number of highly specialized centers with a strong computational background and, so far, such tools have not been implemented into or approved for clinical use. At the same time, efforts are being undertaken to develop user-friendly software which may foster a more pointed search strategy for personalized stimulation settings.

In this study, we used commercially available software tool (lead DBS, matlab) to visualize DBS leads and to simulate potentially effective stimulation settings, that is those resulting in a volume of the electrostatic field located in or within the immediate vicinity of the STN. There was no significant difference in symptom control between the image-based programming and the conventional programming. This finding is consistent with a pilot study including ten PD-patients with octopolar unidirectional DBS which demonstrated equality in motor improvement (21). However, it has obvious advantages in saving programming time and alleviating patients' side effects during programming.

In this study, we could show that image reconstruction techniques may facilitate more targeted testing. We therefore advocate for imaging-based parameters serving as baseline settings (i.e., lead level and directionality) which may be refined based on clinical effects. By this means, the proposed approach or similar techniques may still reduce the total time needed for clinical DBS programming sessions, given the approximate time of 10–20 min at the computer and 20 min with the patient. Particularly, the efforts required for satisfying symptom control may be reduced using image reconstruction initial DBS settings. In general terms, image reconstruction may hence play a role in improving efficiency of DBS programming.

The present study had some limitations, the first of which was its small sample size. Second, this was a retrospective study, and future prospective studies will be designed to investigate the effects of image-reconstruction-guided programming. Third, this study did not determine the long-term effects of DBS in individuals with PD.

In summary, imaging-guided programming of directional DBS leads is possible and leads to save programming time and reduce patient side effects compared with clinical programming. Taking patient-specific anatomy into consideration, this technique or similar approaches may promote more efficient programming of DBS. Given that determination of the lead direction is an indispensable presupposition for successful clinical use of directional DBS, reliable visualization of DBS leads including their rotation angle is possible with image reconstruction with comparable results.

## Data Availability Statement

The raw data supporting the conclusions of this article will be made available by the authors, without undue reservation.

## Ethics Statement

The studies involving human participants were reviewed and approved by Ethics Institutional Committee of the First Hospital Affiliated with USTC (China). The patients/participants provided their written informed consent to participate in this study.

## Author Contributions

JM and BC jointly completed the experiment and the writing. CX and MJ followed up patients. CN took overall control of the whole study. All authors contributed to the article and approved the submitted version.

## Conflict of Interest

The authors declare that the research was conducted in the absence of any commercial or financial relationships that could be construed as a potential conflict of interest.

## Publisher's Note

All claims expressed in this article are solely those of the authors and do not necessarily represent those of their affiliated organizations, or those of the publisher, the editors and the reviewers. Any product that may be evaluated in this article, or claim that may be made by its manufacturer, is not guaranteed or endorsed by the publisher.
